# Synthesis and characterization of activated carbon-supported magnetic nanocomposite (MNPs-OLAC) obtained from okra leaves as a nanocarrier for targeted delivery of morin hydrate

**DOI:** 10.3389/fphar.2024.1482130

**Published:** 2024-10-09

**Authors:** Cem Öziç, Erdal Ertaş, Mehmet Fırat Baran, Ayşe Baran, Elham Ahmadian, Aziz Eftekhari, Rovshan Khalilov, Elvin Aliyev, Mahmut Yıldıztekin

**Affiliations:** ^1^ Department of Basic Medical Sciences, Department of Medical Biology, Kafkas University, Faculty of Medicine, Kars, Türkiye; ^2^ Department of Food Technology, Vocational School of Technical Sciences, Batman University, Batman, Türkiye; ^3^ Department of Biology, Graduate Education Institute, Mardin Artuklu University, Mardin, Türkiye; ^4^ Kidney Research Center, Tabriz University of Medical Sciences, Tabriz, Iran; ^5^ Department of Biochemistry, Faculty of Science, Ege University, Izmir, Türkiye; ^6^ Department of Life Sciences, Western Caspian University, Baku, Azerbaijan; ^7^ Department of Biophysics and Biochemistry, Baku State University, Baku, Azerbaijan; ^8^ Department of Biology and Ecology, Lankaran State University, Lankaran, Azerbaijan; ^9^ Department of Herbal and Animal Production, Koycegiz Vocational School, Mugla Sıtkı Kocman University, Mugla, Türkiye

**Keywords:** morin hydrate, MCF-7, anticancer activity, magnetic nanocomposite, drug delivery

## Abstract

**Introduction:**

The method of encapsulating the drug molecule in a carrier, such as a magnetic nanoparticle, is a promising development that has the potential to deliver the medicine to the site where it is intended to be administered. Morin is a pentahydroxyflavone obtained from the leaves, stems, and fruits of various plantsmainly from the Moraceae family exhibiting diverse pharmacological activities such as anti-inflammatory, anti-oxidant, and free radical scavenging and helps treat diseases such as diabetes, myocardial infarction and cancer.

**Methods:**

In this study, we conducted the synthesis of a nanocomposite with magnetic properties by coating biocompatible activated carbon obtained from okra plant leaves with magnetic nanoparticles.

**Results:**

Characterization of the synthesized activated carbon-coated magnetic nanocomposite was confirmed by Fourier transform infrared, scanning electron microscopy, dynamic light scattering, and zeta potential. The cytotoxic effects of the drug-loaded magnetic nanocomposite were examined in HT-29 (Colorectal), MCF-7 (breast), U373 (brain), T98-G (Glioblastoma) cancer cell lines, and human umbilical vein endothelial cells healthy cell line.

**Discussion:**

We studied the loading and release behavior of morin hydrate in the activated carbon-coated magnetic nanocomposite. Activated carbon-coated magnetic nanocomposite carriers can show promising results for the delivery of Morin hydrate drugs to the targeted site.

## 1 Introduction

Nano-sized materials exhibit a size distribution in the range of 1–100 nm and have a significant surface area, making them highly suitable for biological applications (Adepu and Ramakrishna, 2021; Talha et al., 2022; Mekuye and Abera, 2023). Magnetic nanoparticles (MNPs) have the potential to be used in biomedical settings for purposes like cancer cell imaging, controlled drug release, and hyperthermia treatment. The reason for this is that they have unique physical properties and their ability to interact biologically at the cellular level (Ali et al., 2021; Nasibova et al., 2023). MNPs have several advantageous properties, including their magnetic susceptibility, biocompatibility, stability, and the capacity to be prepared using multiple processes. Moreover, MNPs may be easily manipulated by an external magnetic field, enabling precise control over the release of cancer drugs in a specific location and at a desired rate. Thus, the difficulties encountered in traditional diagnosis and treatment can be overcome (Kush et al., 2021).

Morin hydrate (3,5,7,2′,4′-pentahydroxyflavone) is a yellowish bioflavonoid with versatile biological and pharmacological potential obtained from various plants, especially from the fruits, stems, and leaves of plants belonging to the Moraceae family (Mottaghi and Abbaszadeh, 2021). Many *in vivo* and *in vitro* experiments have reported that morin hydrate (MH) has significant healing potential with a wide range of pharmacological properties including anti-inflammatory, anti-oxidant, free radical scavenger in various diseases such as life-threatening cancer, cardiovascular anomalies, acute lung and liver injury, neuroinflammatory disorders, kidney complications, diabetes, gastritis, through its bioactivity mechanisms (Karamchedu et al., 2020; Balaga et al., 2023).

In this study, activated carbon-coated magnetic nanocomposite (MNPs@OLAC) was synthesized according to the co-precipitation method of interaction of activated carbon (AC) obtained from okra leaves (OL) with magnetic nanoparticles and loaded with MH. MNPs@OLAC magnetic nanocomposite was characterized by Fourier transform infrared spectroscopy (FTIR), scanning electron microscopy (SEM), dynamic light scattering (DLS), and zeta potential techniques. MH release from MNPs@OLAC magnetic nanocomposites under *in vitro* conditions was investigated and analyzed at different pH values and times. Finally, the effects of MH-loaded MNPs@OLAC magnetic nanocomposites on cell viability on the cytotoxicity of HT-29 (Colorectal), MCF-7 (breast), U373 (brain), T98-G (Glioblastoma) cancer cell lines and Human Umbilical Vein Endothelial Cells (HUVEC) healthy cell line were evaluated.

## 2 Materials and methods

### 2.1 Chemicals and reagents

Materials ferric chloride hexahydrate (≥99% purity), ferrous sulfate heptahydrate (≥99% purity), hydrochloric acid (37%, ACS reagent), sodium hydroxide (≥97.0%, pellets), ammonium hydroxide (≥28% purity), Morin hydrate (≥100% purity), methanol (≥99.8%, ACS reagent), dimethyl sulfoxide (DMSO) (≥99.9%, ACS reagent), ethanol (96%, ACS reagent)and zinc chloride (≥98% purity) were purchased from Sigma-Aldrich. All other chemicals used were of analytical grade. Double-distilled water was used for synthesis, solution preparation, and other purposes throughout the experiment.

### 2.2 Characterization

#### 2.2.1 Instruments

The synthesized MNPs@OLAC and MNPs@OLAC-MH were characterized to determine their structural characteristics and morphology using the following methods:

Scanning Electron Microscopy (SEM); Conducted using a QUANTA 400F instrument to examine the surface morphology of MNPs@OLAC and MNPs@OLAC-MH.

Zeta Potential: The stability of nanoparticles in solution was measured using a Zetasizer Nano ZS from Malvern Instruments Ltd. at 25°C.

Laser Particle Sizer; Particle agglomerate size distributions were evaluated using a Mastersizer 2000 Particle Size Analyzer from Malvern Instruments.

Fourier Transform Infrared Spectrophotometry (FT-IR); Functional groups present in MNPs@OLAC and MNPs@OLAC-MH were analyzed using a Perkin Agilent Cary 630 FTIR spectrometer.

UV-Visible Spectrophotometry: The UV–visible absorption spectrum was recorded using an Agilent Cary 60 Spectrophotometer.

Before taking measurements, the samples were obtained from the stock solution by diluting them with pure water as required.

### 2.3 Preparation of OLAC

The leaves of the okra plant were first washed with tap water several times and then washed and cleaned with deionized water 5 times to remove dust and contaminants adhering to the surface of the leaves. After the cleaning process, drying was done in the open air in a fume hood at room temperature, and the dried okra leaves were ground into a powder with the help of an IKA M20 Universal grinder and stored in a container to be used in OLAC production. 10 g of the powder sample obtained by grinding the leaves of the okra plant after drying were weighed, taken into a 500 mL erlenmayer, then, a solution of zinc chloride with a concentration of 1 M (for activation of activated carbon) was added and mixed in a shaking water bath at 80°C for 1 hour (Veerakumar et al., 2016). The precipitate of the mixture, which was brought to room temperature and cooled, was placed in a glass crystallization container to be homogeneous and dried in an oven at 100°C for 24 h. Afterward, the dried mixture was placed in porcelain crucibles and subjected to carbonization in the muffle furnace set at 600°C. The OLAC obtained after the carbonization process was cooled in a desiccator at 25°C. Washing was carried out several times with 0.1 N HCl to remove unreacted zinc, chlorine, and other ions on the surface of OLAC. After washing with HCl, the product was washed with deionized water (until the acidity was removed and the pH value became neutral) and left to dry in an oven at 70°C for 24 h (Farma et al., 2023; Komal et al., 2024).

### 2.4 Preparation of MNPs@OLAC nanocomposite

As reported in the literature, with some modifications, the surface of MNPs was synthesized by coating them with AC obtained from the okra plant (Inbaraj et al., 2021). 3.0 g of iron (III) chloride hexahydrate was dissolved in 50 mL of deionized water in an argon gas atmosphere. In the next step, 2.1 g of iron (II) sulfate heptahydrate was added to the solution containing Fe^3+^ ions and the temperature of the mixture was slowly increased to 90 °C in the presence of argon gas. After 30 min, 10 mL of 26% ammonium hydroxide solution was added to the mixture, and a black solution was formed. This resulting solution continued to be mixed in the magnetic stirrer for another 30 min under the same conditions. Then, 500 mg of OLAC solution dissolved in 100 mL of water was added to the mixture. After the addition of the solution containing OLAC, the product in the magnetic stirrer was mixed for 60 min and the resulting MNPs@OLAC nanocomposite was allowed to cool at room temperature, and the MNPs@OLAC nanocomposite was separated from the solution medium using a magnet. The MNPs@OLAC nanocomposite was washed several times with deionized water and dried in a lyophilizer. After the drying process, the MNPs@OLAC nanocomposite was stored in a colored container to be used in experimental studies.

### 2.5 Investigation of the loading and release conditions of morin hydrate

For the purpose of to loading MH into the MNPs@OLAC magnetic nanocomposite, 10 mL of a 100 μg/mL MH solution dissolved in methanol was added to 50 mg of the nanocomposite. The mixture was then stirred in a water bath at room temperature for 24 hoursThen, the particles were isolated from the solution using an external magnet based on their magnetic properties, and the MH concentration in the solution was measured at a wavelength of 385 nm using UV-Vis spectrophotometry. To calculate drug loading, it was obtained by subtracting the MH concentration in the supernatant from the initial MH concentration using Equation 1. Subsequently, magnetic nanoparticles which contained the medication were isolated by the application of a magnetic field and thereafter underwent a drying procedure.

$$\text{Drug loading efficiency }\left(\%\right)=\frac{\text{Total}\;\text{amount}\;\text{of}\;\text{drug}-\text{Free}\;\text{drug}\;\text{in}\;\text{the}\;\text{supernatant}\;}{\text{Total}\;\text{amount}\;\text{of}\;\text{drug}}\times 100$$ (1)

For the purpose of the MH release investigation, 10 mg of dry drug-loaded nanoparticles were analyzed in 5 mL of PBS with a pH of 5.4 and 7.4 at a temperature of 37°C while being stirred for durations of 1, 3, 6, 12, 18, 24, 36, 48, 60, and 72 h. UV-Vis spectrophotometry was performed at a wavelength of 385 nm to determine the amount of medication that was released after the set incubation periods had passed During the course of the drug release tests, measurements were carried out in accordance with Equation 2.

$$\text{Drug release }\left(\%\right)=\frac{\text{Released}\;\text{drug}}{\text{Total}\;\text{drug}}\times 100$$ (2)

### 2.6 Cell viability test studies

An examination of the viability of the cells was conducted out at the Cell Culture Laboratory of the Dicle University Faculty of Veterinary Medicine Laboratory. The healthy cell line (HUVEC) and the cancer cell lines (MCF-7, U373, T98-G, and HT-29) were obtained from the “American Type Culture Collection” (ATCC).

In accordance with the findings of Baran, the chosen cell lines were grown in a cell culture medium contained within a T75 flask and then incubated at 37°C in an atmosphere containing 5% carbon dioxide (Baran, 2023). After the cells had attained a confluency level of 80%–90%, they were removed from the flasks and the hemocytometric method was used to determine the total number of cells. To carry out two separate time applications, 24 and 48 h, the cells whose numbers were calculated were injected onto 96-well plates in sizes of 10 × 10^5^ in three replicates, with 90 µL of media in each well. Additionally, the cells were inoculated into microplates in triplicate for MNPs@OLAC-MH in pH 7.4 and MNPs@OLAC-MH in pH 5.4 with the intention of doing two separate time applications. The cells were waited for 24 h to adhere to the microplate bottom. The next day, MNPs@OLAC-MH at pH 7.4 and MNPs@OLAC-MH at pH 5.4 were applied to the seeded plates at various concentrations (1, 50, 100, and 200 μg/mL). Liquid with pH 7.4 for MNPs@OLAC-MH and pH 5.4 for MNPs@OLAC-MH was applied to the cells in the control group.

MTT test was conducted at 24 and 48 h following the application, to assess alterations in cell viability. Each well-containing cells in the microplate was supplemented with 10 µL of the prepared MTT solution (5 mg/mL) and then incubated for 3 h at 37°C in a humid atmosphere with 5% CO_2_. Following a 3-h duration, the medium was removed and 100 μL of DMSO were added to each well. After a 20-min incubation period on the shaker, the optical density (OD) values of the wells were measured using UV/Vis Spectrophotometry (Schneider et al., 2021; Montazersaheb et al., 2024).

The average absorbance values obtained by reading the control wells were accepted as 100% live cell values. The absorbance values obtained from the MNPs@OLAC-MH in pH 7.4 and MNPs@OLAC-MH in pH 5.4 applied wells were accepted as % viability by proportioning them to the control absorbance value. MTT trials were repeated three times on different days.

### 2.7 Statistical analysis

The data collected from the study were analyzed using the IBM SPSS 21.0 software package. The statistical significance level was deemed acceptable at a *p*-value of less than 0.05. Data were compared between groups using unpaired t-tests and among multiple groups by one-way ANOVA, followed by Tukey’s *post hoc* tests. *P* < 0.05 was considered statistically significant.

## 3 Results and discussion

### 3.1 FT-IR analysis of MNPs@OLAC and MNPs@OLAC-MH nanocomposites

**FIGURE 1:**
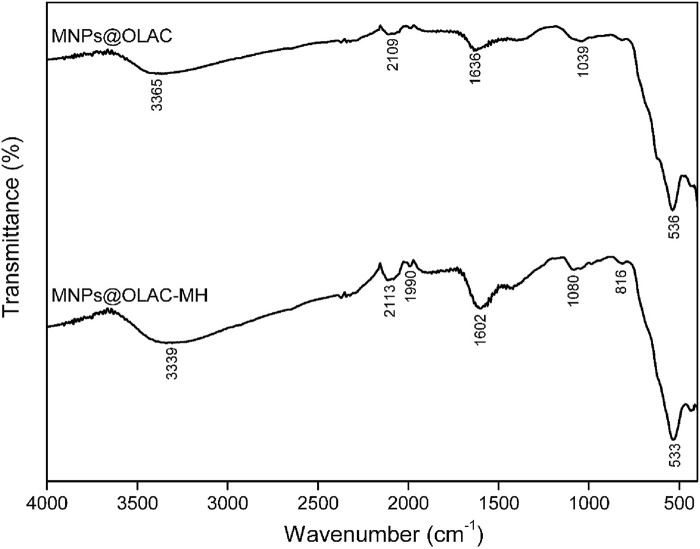
FT-IR spectrometers MNPs@OLAC and MNPs@OLAC@MH.

**FIGURE 2:**
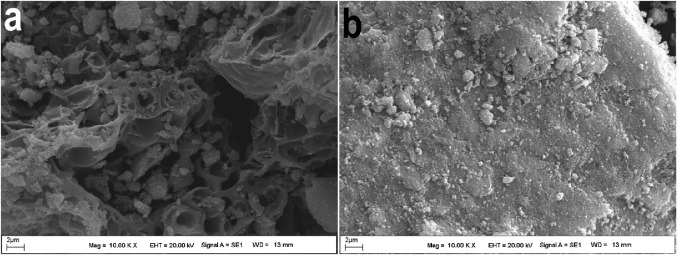
SEM images of **(A)** MNPs@OLAC; **(B)** MNPs@OLAC-MH.

Figure 1 shows the characterization results of MNPs@OLAC and MNPs@OLAC-MH nanocomposites by FTIR spectroscopy. The FT-IR spectrum of MNPs@OLAC nanocomposite at 3365 cm^−1^ is attributed to the stretching and vibration of the–OH (alcoholic and phenolic) group (Ma et al., 2022). When the spectra of MNPs@OLAC and MNPs@OLAC-MH are examined, the peak at 2109 cm^−1^ corresponds to the C≡C stretching of alkynes (Emenike et al., 2023), when the spectrum of MNPs@OLAC and MNPs@OLAC-MH nanocomposites at 2113 cm^−1^ is examined, the measured peak is attributed to the presence of C-H and alkyne groups (Xavier, 2021; Mazumdar et al., 2015; Ferreira et al., 2017) and the peaks observed at 1990–1994 cm^−1^ indicate stretching vibration of C=C bonds (in alkynes) (Al and Kabakcı, 2024). The peaks seen at 1602 and 1636 cm^−1^ (C=O group) correspond to the presence of hydroxyl and carboxylic acid groups (Ezeonuegbu et al., 2021). In addition, for MNPs@OLAC and MNPs@OLAC@MH nanocomposites, the absorption of the peaks at 1039 and 1080 cm^−1^ indicates the presence of C-O and C-N groups (He et al., 2022; Sun et al., 2020). When the spectra of MNPs@OLAC and MNPs@OLAC@MH are examined in Figure 2, the peak seen at 533 and 536 cm^−1^ indicates the presence of Fe-O bond (Tural et al., 2024).

### 3.2 SEM analysis of MNPs@OLAC and MNPs@OLAC-MH nanocomposites

The surface images of MNPs@OLAC and MNPs@OLAC-MH nanocomposites were analyzed using SEM. SEM images of MNPs@OLAC and MNPs@OLAC-MH nanocomposites are shown in Figures 2A, B. The MNPs@OLAC nanocomposite has different pore diameters and a granular surface. The reason for the smoothness on the surface of the MNPs@OLAC nanocomposite after the MNPs@OLAC-MH nanocomposite obtained after the MH drug was bound to the surface of the MNPs@OLAC nanocomposite is due to the irregular distribution of the drug in the surface morphology and pore structures of the nanocomposite (Baig et al., 2022).

### 3.3 DLS analysis of MNPs@OLAC and MNPs@OLAC-MH

**FIGURE 3:**
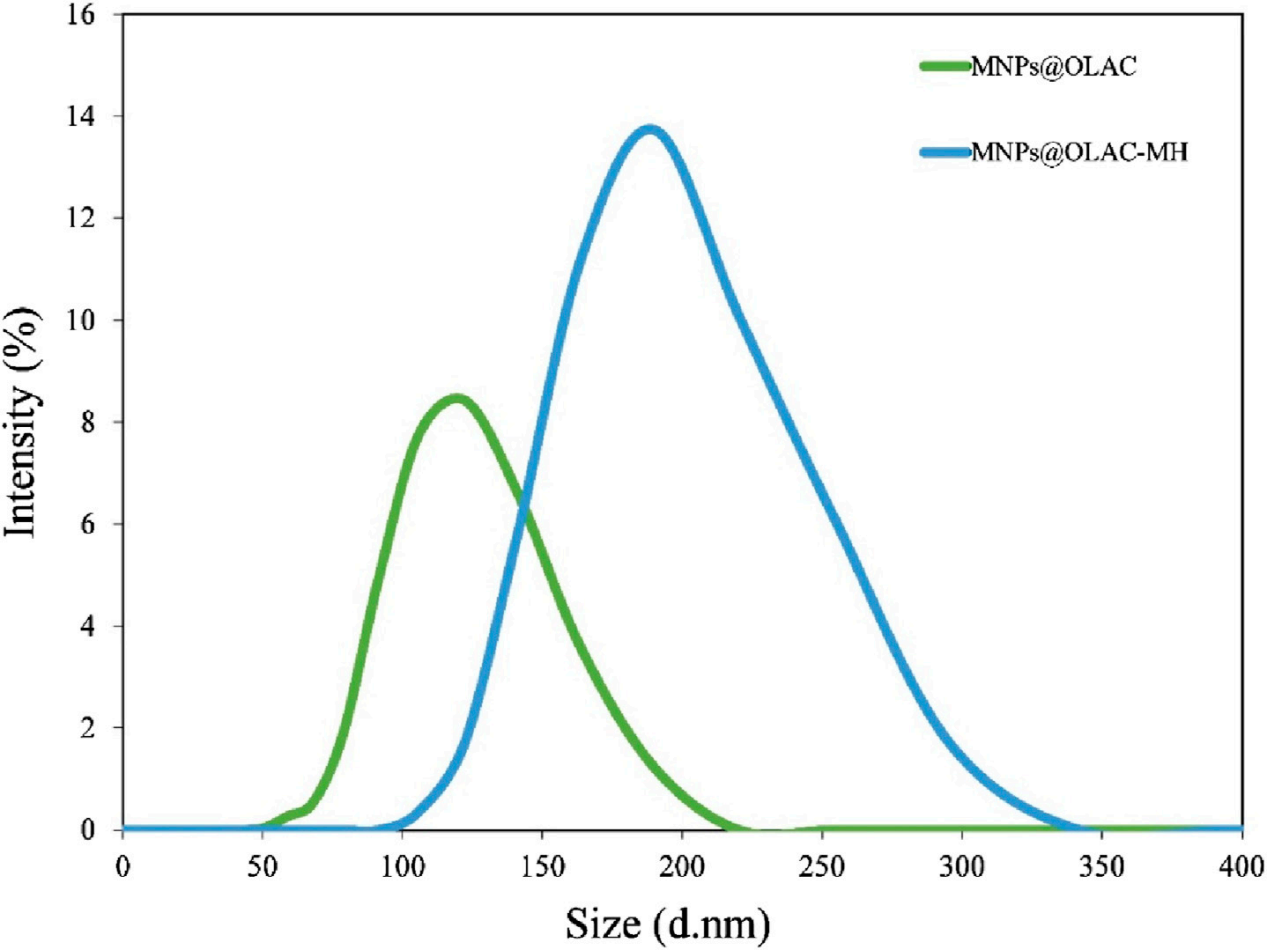
Hydrodynamic diameters of MNPs@OLAC and MNPs@OLAC-MH determined by DLS.

The DLS analysis results of MNPs@OLAC and MNPs@OLAC-MH nanocomposites are shown in Figure 3. Analyses were performed with the solution obtained by weighing 1 mg of the nanocomposite material and sonicating it in 10 mL of deionized water. When the results of the DLS analysis are examined, it is seen that MNPs@OLAC and MNPs@OLAC-MH nanocomposites have an average size distribution of 122 and 190 nm, respectively (Kovrigina et al., 2022).

### 3.4 The zeta-potential measurements of MNPs@OLAC and MNPs@OLAC-MH nanocomposites

**FIGURE 4:**
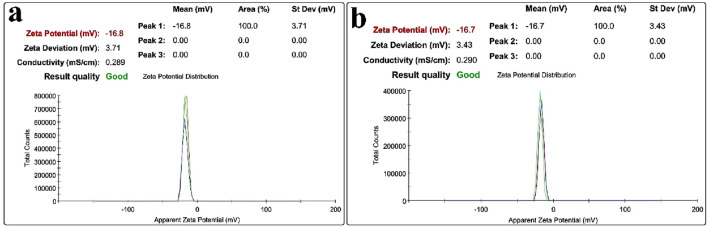
Zeta potential for **(A)** MNPs@OLAC **(B)** MNPs@OLAC-MH.

The zeta potential analysis results of MNPs@OLAC and MNPs@OLAC-MH nanocomposites are shown in Figure 4. As a result of zeta potential measurement, information about the electric charge on the surface of any material is obtained. To prevent agglomeration of MNPs@OLAC and MNPs@OLAC-MH nanocomposites and to ensure stability between nanocomposites, it is desired to obtain either high positive or negative zeta potential values. The potential values of MNPs@OLAC and MNPs@OLAC-MH nanocomposites under the determined optimum conditions were measured as −16.8 and −16.7 mV, respectively. When the results are examined, it is seen that MNPs@OLAC and MNPs@OLAC-MH nanocomposites show low agglomeration due to their negatively charged value, and therefore the prepared suspensions maintain their stability for a long time (Attia et al., 2022).

### 3.5 The behavior of pH-Dependent release

**FIGURE 5:**
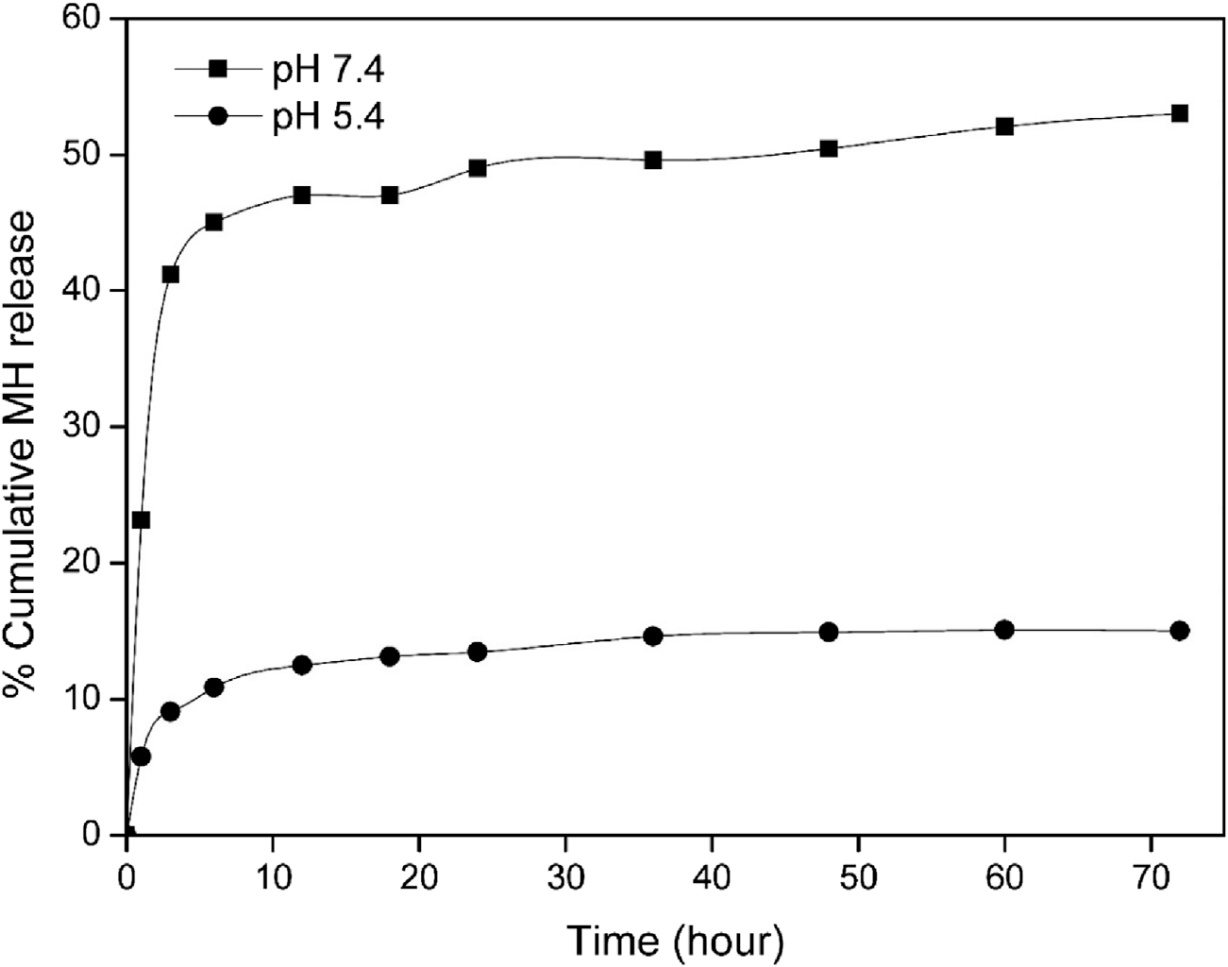
Comparative release profile of MH in MNPs@OLAC conjugated MH.

At a temperature of 37°C, Figure 5 illustrates the cumulative release of MH in MNPs@OLAC at pH 7.4 (the physiological pH) and pH 5.4 (the acidic environment in tumor tissues). MH was found to be released more under physiological settings, and the release of MH changed determined by the pH value that was evaluated. This phenomenon was discovered when different pH values for MH release were investigated (Cunha et al., 2023). To perform the release studies, 10 mL solutions of 96 μg/mL MH loaded into MNPs@OLAC at different pH values and concentrations were used. The ratio of MR mass to the volume of the release medium was found to be 9.6 μg/mL. After 24 h, the release from bulk MH decreased from 48.98% at pH 7.4%–13.47% at pH 5.4. At pH 5.4, in the release of loaded MH in MNPs@OLAC, the release was 10.86% after 6 h, 12.48% after 12 h, 14.89% after 48 h, and 15.02% after 72 h (Kulkarni and Belgamwar, 2019; Jangid et al., 2020). At pH 7.4, in the release of loaded MH in MNPs@OLAC, the release was 45.04% after 6 h, 47.00% after 12 h, 50.44% after 48 h, and 53.01% after 72 h.

MCF-7 breast cancer model (Fuster et al., 2021), U373, HT-29 colon model (Singh et al., 2022), glioblastoma model (Jiang et al., 2024) and T98-G glioblastoma model (Long et al., 2022) cell lines have been widely used as in various studies. Furthermore, HUVEC was utilized as a healthy cell line throughout the course of this research. The MTT assay was utilized in order to examine the cytotoxic effects of MH loaded in MNPs@OLAC nanocomposites against the cell lines HT-29, MCF-7, U373, T98-G, and HUVEC.

**FIGURE 6:**
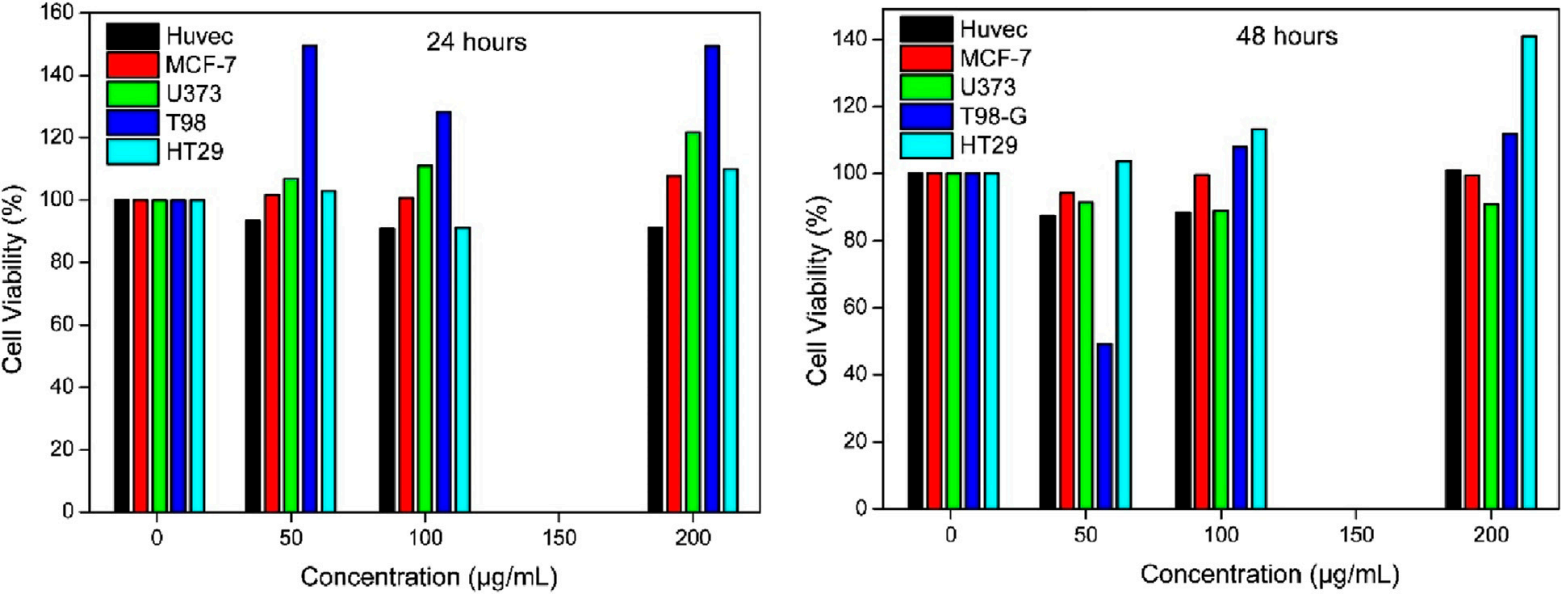
Effect of MNPs@OLAC-MR prepared with PBS 7.4 buffer at concentrations of 1, 50, 100, and 200 μg/mL on cell viability of HUVEC, T98-G, MCF-7, U373, and HT-29 cell lines. Cell viability was evaluated using MTT assay after 24 and 48 h of exposure.

Through the process of reduction, a tetrazolium compound (MTT) is transformed into formazan in the MTT test, which is a colorimetric assay that is extensively used (Lovato et al., 2024). For the purpose of determining cellular metabolic activity, which serves as an indicator of toxicity, colorimetric tests are utilized extensively (Abd AL-Hadi et al., 2024). The effect of MH release in pH 7.4 and 5.4 solutions of MNPs@OLAC-MH prepared in phosphate buffer on cell viability of HT-29, MCF-7, U373, T98-G, and HUVEC cell lines was evaluated after 24 and 48 h. Interaction with MH-loaded MNPs@OLAC nanocomposites at pH 7.4 for 24 h (Figure 6) did not cause significant differences in the viability of tested HT-29, MCF-7, U373, and T98-G cells and was significantly above the concentration ranges of tested MNPs@OLAC-MH for 24 h. However, as seen in Figure 6, after 48 h of interaction, 50 ug/mL concentration and T98-G showed an effect on cell viability.

Effect of MNPs@OLAC-MR (1–200 μg/mL prepared with PBS 7.4 buffer) on cell viability of HUVEC cell line, T98-G cell line, MCF-7 cell line, U373 cell line, and HT-29 cell line. Cell viability was assessed using MTT assay after 24 and 48 h of exposure.

**FIGURE 7:**
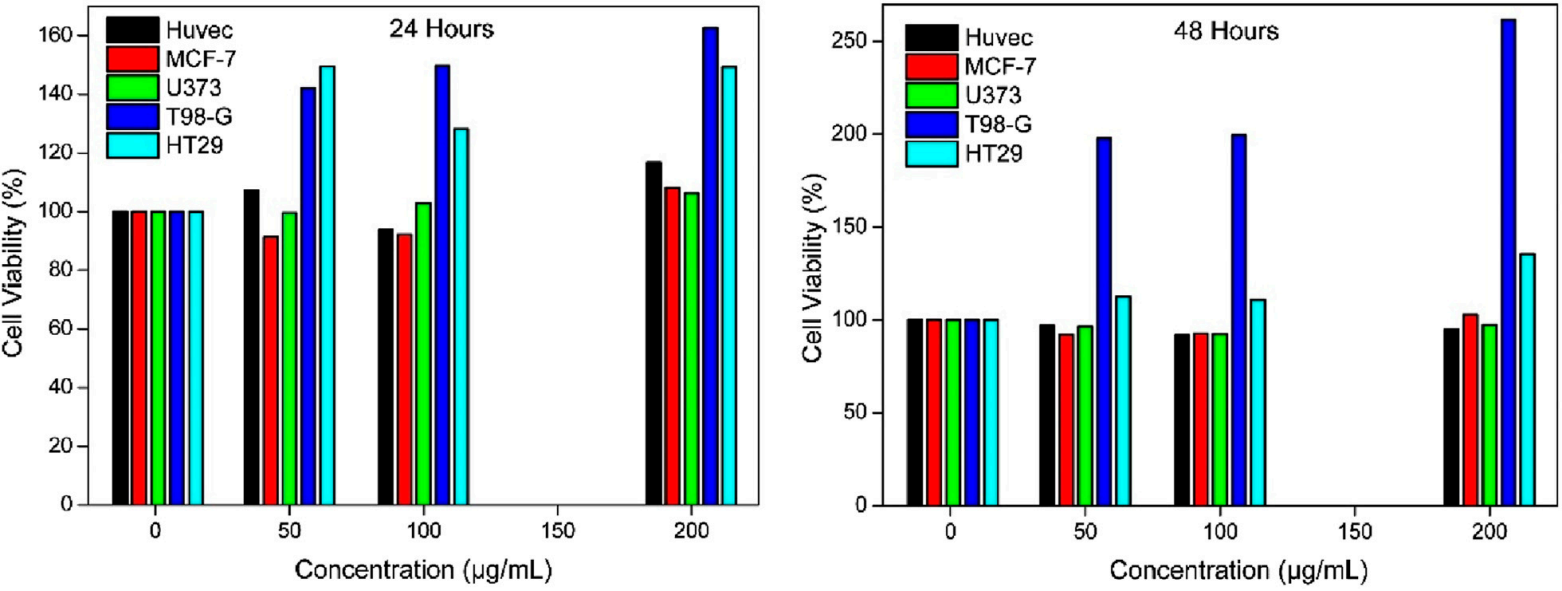
Effect of MNPs@OLAC-MR prepared at concentrations of 1, 50, 100, and 200 μg/mL with PBS 5.4 buffer on cell viability in HUVEC, T98-G, MCF-7, U373, and HT-29 cell lines using MTT assay after 24 and 48 h of exposure.

When exposed to MH-loaded MNPs@OLAC nanocomposites at pH 5.4 for 24 and 48 h, the viability of the evaluated HT-29, MCF-7, U373, and T98-G cells did not show any significant alterations (Figure 7). The concentration ranges of MNPs@OLAC-MH at 24 and 48 h were notably greater than the concentration ranges determined for the tested MNPs@OLAC-MH.

## 4 Conclusion

In this study, we synthesized MNPs@OLAC magnetic nanocomposites that will serve as the transporter of MH, an anticancer drug. MNPs@OLAC nanocomposite was characterized by techniques FTIR, SEM, DLS, and Zeta potential. Morin hydrate was successfully loaded into MNPs@OLAC magnetic nanocomposites using 24 24-h incubation technique. When both pH 5.4 and pH 7.4 were compared, the increase in MH release at pH 7.4 was better for the release studies due to the encapsulation efficiency of MH, possibly due to the effect of increasing both the solubility and dissolution rate of the MH drug due to its transformation into a different form after loading into MNPs@OLAC magnetic nanocomposites. This suggests that MH-loaded MNPs@OLAC magnetic nanocomposites may be a promising nano delivery system to treat HT-29, MCF-7, U373, and T98-G. MNPs@OLAC-MH showed cytotoxic effects on cell viability in T98-G and U373 glioblastoma cell lines, MCF-7 lung cell lines, and HT-29 colon cell lines, both in concentration and time-dependent manner. In particular, T98-G cell lines showed more sensitivity in cell viability compared to U373, MCF-7, and HT-29 cells.

## Data Availability

The original contributions presented in the study are included in the article/supplementary material, further inquiries can be directed to the corresponding author.
